# Association of toll-like receptors polymorphisms with COPD risk in Chinese population

**DOI:** 10.3389/fgene.2022.955810

**Published:** 2022-10-26

**Authors:** Shulei Sun, Yuehao Shen, Jing Feng

**Affiliations:** ^1^ Department of Respiratory and Critical Care Medicine, Tianjin Medical University General Hospital, Tianjin, China; ^2^ Department of Intensive Care Unit, Tianjin Medical University General Hospital, Tianjin, China

**Keywords:** chronic obstructive pulmonary disease, toll-like receptors, polymorphisms, TLR2, TLR3, TLR9

## Abstract

**Background:** Previous studies have reported that the Toll-like receptors (TLRs) are related with the progress of chronic obstructive pulmonary disease (COPD). We aimed to explore the association of TLRs single nucleotide polymorphisms (SNPs) and COPD risk.

**Methods:** 170 COPD patients and 181 healthy controls were enrolled in this case-control study. MassARRAY platform was used for genotyping seven tagging SNPs (*TLR2*: rs3804100, rs4696480, rs3804099; *TLR3*: rs3775290, rs3775291, rs5743305; *TLR9*: rs352140) of TLRs. The correlations between the SNPs and COPD risk were determined using logistic regression.

**Results:** We found that the rs3775291 of *TLR3* significant decreased the risk of COPD (TT *versus* CC: non-adjusted OR = 0.329, 95% CI = 0.123–0.879, *p* = 0.027). In the genetic models analysis, the rs3775291 was associated with a decreased effect of COPD based on the recessive model (TT *versus* CC/CT: non-adjusted OR = 0.377, 95% CI = 0.144–0.988 *p* = 0.047). The rs4696480 of *TLR2* gene was associated with a decreased risk of COPD after adjustment by age and gender (TA *versus* AA: adjusted OR = 0.606, 95% CI = 0.376–0.975, *p* = 0.039).

**Conclusion:** Our study showed that genetic variants in *TLRs* were associated with risk of COPD. The rs3775291 and rs4696480 may act as a potential biomarker for predicting the risk of COPD in Chinese population.

## Introduction

Chronic obstructive pulmonary disease (COPD) is a common inflammatory disease of the airways. (Mathers and Loncar, 2006). COPD is mainly manifested as chronic bronchitis or emphysema. The central feature of COPD is airway remodeling. A large scale epidemiological investigation conducted in China demonstrated that the prevalence of COPD was 8.6% in Chinese aged over 20 years old. The prevalence was 13.7% among people aged over 40 years old. (Wang et al., 2018). The risk factors of COPD including smoking, air pollution and genetic factors. (Lopez-Campos et al., 2016; de Vries et al., 2017; Fakih et al., 2018). Although efforts had been made to explore the potential causes of COPD, the number of COPD patients is increasing. Therefore, a better understanding of the mechanisms resulting in COPD is needed.

Toll-like receptor (TLR) family plays an instructive role in innate immune responses against microbial pathogens, as well as the subsequent induction of adaptive immune responses. (Akira and Takeda, 2004; Kawai and Akira, 2006). Several studies have demonstrated that TLRs have been linked to COPD pathogenesis. For example, TLR2 was decreased in monocytes from patients with COPD. (Droemann et al., 2005). In addition, the TLR3-EGFR signaling pathway was involved in the production of airway remodeling cytokines after virus infection. (Jiang et al., 2016). A recent study showed that miR-149–3 p may increase the inflammatory response in COPD patients through the regulation of the TLR4/NF-κB signaling pathway. (Shen et al., 2017). Another evidence found that the expression of TLR9 (T1237C) is significantly correlated with abnormal response of alveolar macrophages to respiratory pathogens and with severity of COPD. (Berenson et al., 2015). However, little is known about the association between TLRs (*TLR2*, *TLR3*, *TLR9*) polymorphisms and COPD risk in China.

In order to reduce the incidence of COPD, we need to explore the pathogenesis of COPD in China, especially the genetic mechanism. In the current study, we hypothesized that SNPs in TLRs could modulate COPD susceptibility. A case-control study was performed to analyze the association between seven TLRs polymorphisms and the risk of COPD in Chinese population.

## Materials and methods

### Study participants

A total of 170 COPD patients and 181 age matched health controls in Tianjin Medical University General Hospital from January 2019 to November 2021 were recruited in this study. About 2 ml peripheral venous blood was obtained from each participant and immediately stored into tubes containing EDTA for DNA extraction. COPD was defined according to the Global Initiative for Chronic Obstructive Lung Disease (GOLD) criteria. (Vestbo et al., 2013). COPD was defined as post-bronchodilator forced expiratory volume in the first second (FEV_1_)/forced vital capacity (FVC) < 70% and chronic respiratory characteristics include chronic cough, wheezing or dyspnea. Patients were excluded from the study if they had other respiratory diseases such as bronchial asthma, *tuberculosis*, lung cancer or cystic fibrosis. For COPD patients, all the clinical data including age, gender, body mass index, smoking status and lung function was collected through reviewing their medical records. This case-control study was approved by Ethics Committee of Tianjin Medical University General Hospital and conformed to the declarations of Helsinki. Before collecting specimens, the informed consents were written by all participants.

### SNP selection and genotyping analysis

In this study, seven SNPs in TLRs (*TLR2*: rs3804100, rs4696480, rs3804099; *TLR3*: rs3775290, rs3775291, rs5743305; *TLR9*: rs352140) were selected. All of the SNPs had minor allele frequency (MAF) 10% in global population from the 1000 Genome Project, and these SNPs locate in different functional regions, such as promoter and exon. Genomic DNA was isolated from peripheral blood using DNA extraction kit (TianGen biotechnology, Beijing, China), and concentration was detected by the NanoDrop 1000(Thermo Fisher Scientific, Waltham, MA, United States). Genotyping was done by Sangon Biotech Company (Shanghai, China) using Sequenom MassARRAY platform (San Diego, CA, United States) according to the protocol. The primers used for the seven SNPs were shown in Table 1.

**TABLE 1:** Primers used for this study.

SNP ID	First PCR primer	Second PCR primer	UEP SEQ
rs3804100	ACG​TTG​GAT​GGT​CAG​TGG​CCA​GAA​AAG​ATG	ACG​TTG​GAT​GTT​CCA​GTG​TCT​TGG​GAA​TGC	TCC​AGC​ACA​CGA​ATA​CAC​AG
rs4696480	ACG​TTG​GAT​GCT​CAC​CAT​GTG​ATG​CTT​TCC	ACG​TTG​GAT​GAG​TCC​AAG​ATT​GAA​GGG​CTG	AGCCAGATGACCCTC
rs3804099	ACG​TTG​GAT​GCT​GCT​TCA​TAT​GAA​GGA​TCA​G	ACG​TTG​GAT​GGA​TCT​ACA​GAG​CTA​TGA​GCC	gAT​GAA​GGA​TCA​GAT​GAC​TTA​C
rs3775290	ACG​TTG​GAT​GCT​GCA​GGT​ACT​TGT​TGT​AGG	ACG​TTG​GAT​GAT​TGG​GCA​AGA​ACT​CAC​AGG	TTG​TTG​TAG​GAA​AGA​TAG​ATT​TC
rs3775291	ACG​TTG​GAT​GGT​ATC​ACT​TGC​TCA​TTC​TCC	ACG​TTG​GAT​GCC​CAA​CCA​AGA​GAA​AGC​ATC	TCA​TTC​TCC​CTT​ACA​CAT​A
rs5743305	ACG​TTG​GAT​GAT​CAG​AGA​CAT​GTA​GCC​CTG	ACG​TTG​GAT​GGA​AAC​AAG​CGT​GCA​TCA​TGG	AGC​CCA​GTA​ACT​ATA​AAG​CGG
rs352140	ACG​TTG​GAT​GTG​GCT​GTT​GTA​GCT​GAG​GTC	ACG​TTG​GAT​GAT​AAG​CTG​GAC​CTC​TAC​CAC	ctt​cCC​AGG​GCC​TCC​AGT​CG

UEP SEQ, unextended mini-sequencing primer.

### Statistical analysis

Hardy-Weinberg equilibrium test was performed on the control group using the χ^2^ test to evaluate the reliability of the control group. Allele frequencies and genotype frequencies and differences in the clinical characteristics between the two groups were analyzed with Student’s t test or χ^2^ test. The association of each SNP and COPD susceptibility was estimated by using unconditional logistic regression analyses with odds ratios (ORs) and 95% confidence intervals (CIs). Four models (dominant model, recessive model, heterozygote comparison and homozygote comparison) were used to assess the association between each genotype and the risk of COPD. *p* < 0.05 were considered statistically significant. All statistical analysis was performed using SPSS software version 20 (SPSS, Chicago, IL, United States).

## Results

### Clinical characteristics of the study subjects

The basic clinical characteristics of the case and control groups are described in Table 2. A total of 170 COPD cases (118 men and 52 women; mean age, 66.62 ± 6.64 years) and 181 controls (114 men and 67 women; mean age, 65.52 ± 4.83 years) were included in the study. There were no significant differences in age, gender and body mass index between the case and control groups (*p* > 0.05). However, there were significant differences in smoking status between the two groups (*p* < 0.05). The pulmonary function parameters of COPD group (FEV_1_/FVC, 50.52 ± 11.6) met the inclusion criteria of GOLD.

**TABLE 2:** Characteristics of cases and controls in this study.

Variables	Case (n = 170)	Control (n = 181)	p-value
Age (Mean ± SD)	66.62 ± 6.64	65.52 ± 4.83	0.074
Gender (Male/Female)	118/52	114/67	0.204
Smoking status			
Smoker/Nonsmoker	132/38	90/91	<0.001
BMI (Mean ± SD)	24.05 ± 4.25	24.15 ± 3.85	0.814
Lung function (Mean ± SD)			
FEV1 (%pre)	44.81 ± 18.93		
FEV1/FVC (%)	50.52 ± 11.60		

**Abbreviations:** n, number; BMI, body mass index; SD, standard deviation.

### HWE and SNPs alleles

The detailed information of candidate SNPs in TLRs (TLR2, TLR3, TLR9) are shown in Table 3. These SNPs are mainly locate in the exons, introns and promoter regions of genes. The MAF of the SNPs in the control group was similar to those reported for the 1000 Genome Project. The genotypes distributions of all SNPs among the control group were in accordance with Hardy-Weinberg equilibrium (*p* > 0.05).

**TABLE 3:** Characteristics of the SNPs in this study

SNP ID	Gene	Position	Role	Alleles A/B	MAF		p-value HWE
					1000G	Control	
rs3804100	TLR2	4q31.3	Exon	T/C	0.108	0.323	0.095
rs4696480	TLR2	4q31.3	Intron	A/T	0.578	0.436	0.176
rs3804099	TLR2	4q31.3	Exon	T/C	0.415	0.354	0.132
rs3775290	TLR3	4q35.1	Exon	C/T	0.272	0.276	0.054
rs3775291	TLR3	4q35.1	Exon	C/T	0.231	0.307	0.722
rs5743305	TLR3	4q35.1	Promoter	T/A	0.323	0.293	0.108
rs352140	TLR9	3p21.2	Exon	C/T	0.415	0.456	0.188

**Abbreviations:** MAF, minor allele frequency; HWE, Hardy–Weinberg equilibrium.

### Associations of selected SNPs in TLR genes and COPD risk

The genotype frequencies of the TLRs (TLR2, TLR3, TLR9) polymorphisms are presented in Table 4. When considering the rs3775291 SNP in the TLR3 gene, compared with the CC genotype, the TT genotype significantly decreased the risk of COPD (non-adjusted OR = 0.329, 95% CI = 0.123–0.879, *p* = 0.027). Furthermore, we used four genetic models (dominant, recessive, heterozygote and homozygote) to analyze the association between the rs3775291 and risk of COPD. The results showed that the rs3775291 TT genotype was associated with a decreased risk of COPD based on the recessive model (non-adjusted OR = 0.377, 95% CI = 0.144–0.988, *p* = 0.047) (Table 5).

**TABLE 4:** Genotypes frequencies of the SNPs and their associations with risk of COPD

SNP ID	Genotype	Genotype frequencies		Without adjustment	With adjustment		
		Case n (%)	Control n (%)	OR (95%CI)	p-value	OR (95%CI)	P-value^a^
Total N		170	181				
rs3804100	TT	70 (41.2)	78 (43.1)	1			
	TC	84 (49.4)	89 (49.2)	1.052 (0.678–1.632)	0.822	1.086 (0.697–1.694)	0.714
	CC	16 (9.4)	14 (7.7)	1.273 (0.580–2.796)	0.547	1.322 (0.595–2.941)	0.493
rs4696480	AA	66 (38.8)	53 (29.3)	1			
	TA	77 (45.3)	98 (54.1)	0.631 (0.395–1.008)	0.054	0.606(0.376–0.975)	0.039
	TT	27 (15.9)	30 (16.6)	0.723 (0.384–1.361)	0.315	0.715 (0.378–1.354)	0.303
rs3804099	TT	68 (40.0)	71 (39.2)	1			
	TC	84 (49.4)	92 (50.8)	0.953 (0.611–1.488)	0.833	0.969 (0.618–1.517)	0.889
	CC	18 (10.6)	18 (10.0)	1.044 (0.502–2.173)	0.908	1.136 (0.537–2.405)	0.738
rs3775290	CC	77 (45.3)	100 (55.2)	1			
	CT	71 (41.8)	62 (34.3)	1.487 (0.946–2.337)	0.085	1.524 (0.966–2.405)	0.070
	TT	22 (12.9)	19 (10.5)	1.504 (0.760–2.974)	0.241	1.516 (0.759–3.026)	0.238
rs3775291	CC	98 (57.7)	86 (47.5)	1			
	CT	66 (38.8)	79 (43.7)	0.733 (0.474–1.135)	0.164	0.697 (0.447–1.087)	0.112
	TT	6 (3.5)	16 (8.8)	0.329(0.123–0.879)	0.027	0.324(0.121–0.871)	0.026
rs5743305	TT	89 (52.4)	95 (52.5)	1			
	TA	69 (40.6)	66 (36.5)	1.116 (0.716–1.740)	0.629	1.127 (0.719–1.765)	0.602
	AA	12 (7.0)	20 (11.0)	0.640 (0.296–1.386)	0.258	0.630 (0.288–1.377)	0.247
rs352140	CC	61 (35.9)	58 (32.0)	1			
	CT	72 (42.3)	81 (44.8)	0.845 (0.523–1.365)	0.492	0.884 (0.543–1.441)	0.622
	TT	37 (21.8)	42 (23.2)	0.838 (0.474–1.481)	0.542	0.862 (0.485–1.534)	0.614

**Notes:** a *p*-values were calculated by unconditional logistic regression adjusted for age and gender.

**Abbreviations:** OR, odds ratio; CI, confidence interval.

**TABLE 5:** Association between rs3775291 and risk of COPD in different inheritance models.

SNP ID	Genotype	Genotype	Frequencies	Without adjustment		With adjustment^a^	
		Case n (%)	Control n (%)	OR (95%CI)	p-value	OR (95%CI)	P-value^a^
	CC	98 (57.7)	86 (47.5)	1		1	
Heterozygote	CT	66 (38.8)	79 (43.7)	0.733 (0.474–1.135)	0.164	0.697 (0.447–1.087)	0.112
Homozygote	TT	6 (3.5)	16 (8.8)	0.329(0.123–0.879)	0.027	0.324(0.121–0.871)	0.026
Dominant	CC	98 (57.6)	86 (47.5)	1		1	
	CT + TT	72 (42.4)	95 (52.5)	0.665 (0.436–1.014)	0.058	0.661 (0.433–1.010)	0.056
Recessive	CC + CT	164 (96.5)	165 (91.2)	1		1	
	TT	6 (3.5)	16 (8.8)	0.377(0.144–0.988)	0.047	0.394 (0.150–1.034)	0.059

**Notes:** ^a^ *p*-values were calculated by unconditional logistic regression adjusted for age and gender.

**Abbreviations:** OR, odds ratio; CI, confidence interval.

All gene polymorphisms susceptibility analysis of COPD was performed after adjustment by age and gender. For the rs3775291 SNP, TT genotype decreased the risk of COPD in the homozygote comparison model (adjusted OR = 0.324, 95% CI = 0.121–0.871, *p* = 0.026) (Table 4). Furthermore, the results demonstrated that the rs4696480 in TLR2 was associated with a decreased risk of COPD based on heterozygote comparison model (adjusted OR = 0.606, 95% CI = 0.376–0.975, *p* = 0.039) (Table 4).

## Discussion

In the present case-control study, we investigated the potential association of TLRs (TLR2, TLR3, TLR9) polymorphisms with COPD risk in Chinese population. Our results revealed that the rs3775291 and rs4696480 significant decreased the risk of COPD. The rs3775291 is a missense mutation located in the exon of *TLR3*, and its change may affect the protein structure of TLR3. In addition, rs4696480 is located in the intron of *TLR2*, and its change may affect the protein expression of TLR2. Based on the recessive and homozygote model, the SNP rs3775291 was associated with a decreased risk of COPD and this association was model dependent. Two previous studies have reported the association between TLR2 polymorphisms and COPD risk in Caucasus population. One study found that *TLR2* polymorphisms have no effect on disease severity of COPD in Greek population. (Apostolou et al., 2017). However, another study from Netherlands showed that *TLR2* polymorphism was related to the decline of pulmonary function in COPD patients. (Budulac et al., 2012). The difference in their results may be due to the differences in genetic background and gene frequency between populations in different countries. Therefore, it is necessary to explore the association between TLRs polymorphisms and COPD risk in Chinese population.

Other researchers have also explored the relationship between gene polymorphisms and COPD susceptibility. A study from Egypt showed that rs1051730 of *CHRNA3* might be a risk factor of COPD. (El et al., 2020). Karimi et al. observed that the rs1042713 in *ADRB2* was associated with a reduced risk of COPD exacerbation in patients of inhaled β2-agonists. Karimi et al. (2019) Moreover, another study found that *FGFR2* and *MGAT5* genetic polymorphisms were correlated with the risk of COPD in the Chinese people. (Li et al., 2021). Zhang et al. suggested that a new variant rs17014601 of *FAM13A* could increase the risk of COPD in Chinese population. Zhang et al. (2018) Base on above results and studies, we speculated that gene polymorphisms play an important role in the pathological process of COPD.

In the present study, we explored seven SNPs in TLRs, including rs3804100, rs4696480, rs3804099, rs3775290, rs3775291, rs5743305, rs352140. Some gene polymorphisms included in our study have also been reported in other diseases. For instance, one study showed that rs4696480 of *TLR2* may have significant effects on the heritability of psoriasis in the Turkish population. (Sabah-Özcan and Gürel, 2019). Moreover, the rs3775290 of *TLR3* might be a protective factor for sporadic parkinson’s disease in Han Chinese population. (Wang et al., 2020). Recent evidence suggest that *TLR9* rs352140 was associated with early-stage cervical cancer. (Pandey et al., 2019). The risk of multiple lung diseases can also be affected by these included gene polymorphisms. Smit et alreported that *TLR2* rs3804099 was associated with an increased risk for asthma in family-based analyses. Smit et al. (2009) One study from Germany found that *TLR2* rs3804099 may affect the risk of pulmonary *tuberculosis* in Moldavian population. (Varzari et al., 2019). As far as we know, we are the first to report the association between TLRs polymorphisms and COPD risk in Chinese population.

The downstream genes of TLR family signaling pathway are various inflammatory cytokines including TNF-α, IL-6 and IL-10. (Leifer and Medvedev, 2016; Luo et al., 2019). Different inflammatory cytokines play an important role in the pathological process of COPD. For example, one research suggested that miR-378 inhibited the development of smoking-induced COPD by targeting TNF-α. (Zhang et al., 2019). Another evidence found that lung fibroblasts participated in the chronic inflammation in COPD by releasing IL-6 and IL-8. (Zhang et al., 2012). Silva et al. showed that IL-10 expression was associated with the severity of COPD. Silva et al. (2018) In lung cancer, a variety of drugs targeting EGFR mutations have been developed. (Remon et al., 2018). However, there are still few targeted drugs for COPD. Based on the above studies, we speculate that TLRs polymorphisms may be potential therapeutic target for COPD.

The main innovation of our study is that rs3775291 and rs4696480 of TLRs may be potential biomarkers for predicting COPD risk in Chinese population. However, there are several limitations of this case-control study. First, the statistical power of this study may be restricted by the small sample size. The larger populations are needed to confirm our results. Second, the biological function and mechanism of rs3775291 and rs4696480 in COPD has not been explored. Therefore, the detailed mechanism of TLR gene polymorphisms affecting the biological function of COPD needs to be explored in the future. Third, there are differences in smoking status between the case group and the control group, which may affect our analysis results. We believe that a larger sample of research is needed to explore this problem in the future.

## Conclusion

In summary, our study showed that genetic variants in *TLRs* were associated with risk of COPD. The rs3775291 and rs4696480 may act as a potential biomarker for predicting the risk of COPD in Chinese population. Further studies are required to clarify the role of rs3775291 and rs4696480 in development of COPD.

## Data Availability

The original contributions presented in the study are included in the article/supplementary material, further inquiries can be directed to the corresponding author.
